# Molecular Survey and Genetic Characterization of *Anaplasma marginale* in Ticks Collected from Livestock Hosts in Pakistan

**DOI:** 10.3390/ani12131708

**Published:** 2022-07-01

**Authors:** Zaibullah Khan, Shehla Shehla, Abdulaziz Alouffi, Muhammad Kashif Obaid, Alam Zeb Khan, Mashal M. Almutairi, Muhammad Numan, Ome Aiman, Shumaila Alam, Shafi Ullah, Sher Zaman Safi, Tetsuya Tanaka, Abid Ali

**Affiliations:** 1 Department of Zoology, Abdul Wali Khan University Mardan, Mardan 23200, Pakistan; zaib.zoologist@gmail.com (Z.K.); habibshehla2018@gmail.com (S.S.); kashifobaidkanz@gmail.com (M.K.O.); mn.awkum@gmail.com (M.N.); omeaiman1@gmail.com (O.A.); haidermurad1@yahoo.com (S.A.); shafi_ullah@awkum.edu.pk (S.U.); 2 King Abdulaziz City for Science and Technology, Riyadh 12354, Saudi Arabia; asn1950r@gmail.com; 3 Department of Pediatrics, Yale School of Medicine, Yale University, New Haven, CT 06510, USA; alamzeb.khan@yale.edu; 4 Department of Pharmacology and Toxicology, College of Pharmacy, King Saud University, Riyadh 11451, Saudi Arabia; mmalmutairi@ksu.edu.sa; 5 Faculty of Medicine, Bioscience and Nursing, MAHSA University, Jenjarom 42610, Selangor, Malaysia; dr.szsafi@cuilahore.edu.pk; 6 Interdisciplinary Research Centre in Biomedical Materials, Lahore Campus, COMSATS University Islamabad, Lahore 54000, Pakistan; 7 Laboratory of Infectious Diseases, Joint Faculty of Veterinary Medicine, Kagoshima University, 1-21-24 Korimoto, Kagoshima 890-0065, Japan

**Keywords:** *Anaplasma marginale*, cattle, livestock, Pakistan, ticks

## Abstract

**Simple Summary:**

Ticks transmit different disease-causing agents to humans and animals. Pakistan is an agricultural country, the rural economy mainly relies on livestock farming, and tick infestation is a severe constraint to its livelihood. The genus *Anaplasma* comprises obligate Gram-negative intracellular bacteria multiplying within the host cells and can be transmitted to humans and animals through the tick vector. The current study aimed to molecularly characterize the *Anaplasma* spp. in hard ticks infesting livestock in different districts of Khyber Pakhtunkhwa, Pakistan. The present study reported nine species of hard ticks infesting different hosts. The most prevalent tick life stage was adult females, followed by nymphs and adult males. In the phylogenetic tree, *16S rDNA* sequences of *Anaplasma* spp. clustered with sequences of *A. marginale*. The hard ticks act as a carrier for the transmission of *A. marginale*. Further extensive country-wide research is required to explore the diverse tick species and the associated pathogens in Pakistan.

**Abstract:**

Ticks transmit pathogens to animals and humans more often than any other arthropod vector. The rural economy of Pakistan mainly depends on livestock farming, and tick infestations cause severe problems in this sector. The present study aimed to molecularly characterize the *Anaplasma* spp. in hard ticks collected from six districts of Khyber Pakhtunkhwa, Pakistan. Ticks were collected from various livestock hosts, including cattle breeds (Holstein-Friesian, Jersey, Sahiwal, and Achai), Asian water buffaloes, sheep, and goats from March 2018 to February 2019. Collected ticks were morphologically identified and subjected to molecular screening of *Anaplasma* spp. by amplifying *16S rDNA* sequences. Six hundred seventy-six ticks were collected from infested hosts (224/350, 64%). Among the nine morphologically identified tick species, the highest occurrence was noted for *Rhipicephalus microplus* (254, 37.6%), followed by *Hyalomma anatolicum* (136, 20.1%), *Rhipicephalus haemaphysaloides* (119, 17.6%), *Rhipicephalus turanicus* (116, 17.1%), *Haemaphysalis montgomeryi* (14, 2.1%), *Hyalomma dromedarii* (11, 1.6%), *Haemaphysalis bispinosa* (10, 1.5%), *Hyalomma scupense* (8, 1.2%), and *Haemaphysalis kashmirensis* (8, 1.2%). The occurrence of tick females was highest (260, 38.5%), followed by nymphs (246, 36.4%) and males (170, 25.1%). Overall, the highest occurrence of ticks was recorded in the Peshawar district (239, 35.3%), followed by Mardan (183, 27.1%), Charsadda (110, 16.3%), Swat (52, 7.7%), Shangla (48, 7.1%), and Chitral (44, 6.5%). Among these ticks, *Anaplasma marginale* was detected in *R. microplus*, *R. turanicus*, and *R. haemaphysaloides*. The *16S rDNA* sequences showed high identity (98–100%) with *A. marginale* reported from Australia, China, Japan, Pakistan, Thailand, Uganda, and the USA. In phylogenetic analysis, the sequence of *A. marginale* clustered with the same species reported from Australia, China, Pakistan, Thailand, Uruguay, and the USA. Further molecular work regarding the diversity of tick species and associated pathogens is essential across the country.

## 1. Introduction

Ticks (Acari: Ixodoidea) are most prevalent in the tropical and subtropical regions of the world, parasitizing almost all terrestrial and semi-aquatic vertebrates [1]. Ticks can transmit various pathogens to their vertebrate hosts, including viruses, bacteria, and protozoans [2,3]. Tick-borne pathogens (TBPs) that often infect animals include *Babesia* spp., *Anaplasma* spp., *Theileria* spp., *Ehrlichia* spp., and *Rickettsia* spp. [2,4,5]. Anaplasmosis, theileriosis, babesiosis, and cowdriosis are the leading tick-borne diseases (TBDs). They affect bovines and small ruminants. Among these, the first three diseases have a prime impact on the economy of Pakistan [6,7].

*Anaplasma* species (Anaplasmataceae: Rickettsiales) are obligate intracellular Gram-negative bacteria transmitted by ticks to animal hosts, including humans, that can propagate and survive within the host cells [8,9,10]. Tick genera such as *Rhipicephalus*, *Haemaphysalis*, *Dermacentor*, *Amblyomma*, and *Ixodes* transmit *Anaplasma* spp. to different hosts [11]. *Anaplasma marginale* is an important bacterium of public and veterinary health that is mainly distributed in tropical, subtropical, and temperate regions [11,12]. The tick genera known for transmitting various TBPs in Pakistan are *Rhipicephalus*, *Hyalomma*, *Haemaphysalis*, *Ixodes*, *Ornithodoros*, and *Argas* [5,13,14,15,16,17,18]. Anaplasmosis is one of the most common hemo-rickettsiales diseases of cattle and Asian water buffaloes [19], and various *Rhipicephalus* and *Hyalomma* species transmit *Anaplasma* spp., such as *Anaplasma centrale*, *Anaplasma ovis*, *Anaplasma* platys-like organism, and *A. marginale* in Pakistan [6,19,20].

In Pakistan, the Khyber Pakhtunkhwa (KP) province is a hotspot for developing and reoccurring TBDs of veterinary and public health relevance [5,6,15,16,18,21]. Due to the extension in dairy cattle industries and the introduction of exotic cattle breeds, the surveillance of ticks and TBPs in indigenous and exotic cattle breeds is essential. There is a scarcity of available information regarding the detection of *Anaplasma* spp. in ticks [5,19]. The current study aimed to investigate different tick species to detect *Anaplasma* spp. throughout KP.

## 2. Materials and Methods

### 2.1. Ethical Approval

The research study was approved by the Advanced Studies Research Board (ASRB) members of Abdul Wali Khan University Mardan, Pakistan (Dir/A&R/AWKUM/2018/1410). Verbal/oral permission was taken from the livestock owners and farmers during tick collection.

### 2.2. Study Area

**Figure 1:**
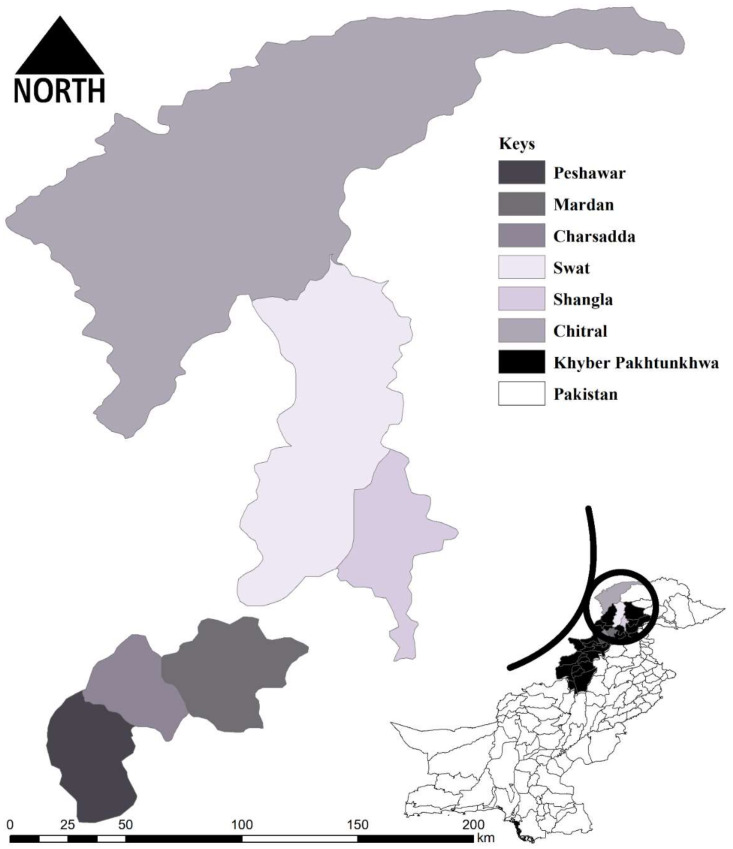
Map showing study districts where tick specimens were collected.

Tick specimens were collected from various herds randomly in six districts, including Peshawar (34.039825° N, 71.566832° E), Mardan (34.194697° N, 72.050557° E), Charsadda (34.161297° N, 71.753660° E), Swat (35.2227° N, 72.4258° E), Shangla (34.8883° N, 72.6003° E), and Chitral (35.7699° N, 71.7741° E) of KP, Pakistan. Ticks collected from hosts in the northern three districts, including Chitral, Shangla, and Swat, were associated with transhumant herds. The average temperature of northern districts (Chitral, Shangla, and Swat) are 0–15 °C and 15–35 °C, while the southern districts (Peshawar, Mardan, and Charsadda) are 10–28 °C and 30–43 °C in winter and summer seasons, respectively (climate-data.org) (accessed on 26 April 2022). Tick specimens were collected from various hosts available in accessible regions of the study area. The geographical coordinates of each collection site were obtained using a Global Positioning System (GPS) and processed in Microsoft Excel V. 2016 (Microsoft 365^®^), and then imported to ArcGIS V. 10.3.1 (ESRI, Redlands, CA, USA) to design the map (Figure 1).

### 2.3. Ticks Collection and Preservation

Tick specimens were randomly collected from March 2018 to February 2019 during four seasons (spring, summer, fall, and winter). These specimens were collected from livestock hosts having different ages, genders, and host types reared at houses (herd size at house was 1–4 animals) and animal farms (herd size at farm was 20–80 animals). The collections were randomly done from different cattle breeds (Holstein-Friesian, Jersey, Sahiwal, and Achai), Asian water buffaloes, sheep, and goats at various collection sites in the six districts of KP, Pakistan. Ticks were washed with distilled water followed by 70% ethanol to remove the contaminants and tissues from the tick’s body. Finally, the specimens were preserved in 100% ethanol for further experimental work.

### 2.4. Morphological Identification of Ticks

The collected tick specimens were identified under a stereo zoom microscope (SZ61, Olympus, Tokyo, Japan) using standard taxonomic keys based on morphological features [22,23,24,25,26,27].

### 2.5. DNA Extraction and PCR

Genomic DNA was extracted from 268 selected ticks (Table 1) using the phenol-chloroform method [28]. The extracted genomic DNA samples were quantified via NanoQ (Optizen, Daejeon, South Korea) and stored at −20 °C for further analysis. A conventional PCR (GE-96G, BIOER, Hangzhou, China) was performed to amplify the *16S rRNA* gene for *Anaplasma* spp. using a pair of primers (Ehr-F2, 5′-AGA GTT TGA TCC TGG CTC AG-3′ and Ehr-R, 5′-GAG TTT GCC GGG ACT TYT TCT-3′) [29]. PCR reaction mixture of 25 µL was comprised of 2 µL genomic DNA (50 ng), 1 µL each forward and reverse primer (10 µM), 8.5 µL PCR water, and 12.5 µL DreamTaq PCR Master Mix (2×) (Thermo Fisher Scientific, Inc., Waltham, MA, USA). Thermocycling conditions were: 95 °C for 3 min, followed by 35 cycles at 95 °C for 30 s, 50 °C for 30 s, 72 °C for 1 min, and final extensions at 72 °C for 7 min. In each PCR reaction, PCR water and *Rickettsia massiliae* DNA were taken as negative and positive controls, respectively. The PCR products were run on a 2% agarose gel electrophoresis and observed via Gel Documentation (BioDoc-It™ Imaging Systems UVP, LLC, Upland, CA, USA). The amplicons were purified through the DNA Clean Kit and Concentrator (Zymo Research, Irvine, CA, USA).

### 2.6. DNA Sequencing and Phylogenetic Analysis

Amplified amplicons of *16S rDNA* of *Anaplasma* spp. from different tick species infesting Holstein-Friesian, Jersey, and Asian water buffaloes were sequenced in both directions (Macrogen, Inc., Seoul, South Korea). The obtained sequences were trimmed to remove the contaminated and poor reading regions via SeqMan V. 5 (DNASTAR, Inc., Madison, WI, USA). The obtained sequences were subjected to the Basic Local Alignment Search Tool (BLAST) [30] at National Center for Biotechnology Information (NCBI). The homologous sequences were downloaded in FASTA format from NCBI based on their high percentage identity. The downloaded sequences were aligned with the obtained sequences and an outgroup sequence using ClustalW multiple alignments [31] in BioEdit alignment editor V.7.0.5 (Raleigh, NC, USA) [32]. The phylogenetic tree was constructed through the Maximum-Likelihood statistical method and Tamura-Nei model [33] with a 1000 bootstrapping value in Molecular evolutionary genetics analysis (MEGA-X) [34].

### 2.7. Statistical Analyses

The descriptive statistical analyses were performed in Microsoft Excel V. 2016 (Microsoft 365^®^). Chi-square tests were performed in the GraphPad Prism V. 5 (GraphPad Software, Inc., San Diego, CA, USA), and the analysis was considered significant at a *p*-value < 0.05.

## 3. Results

### 3.1. Ticks Description

A total of 350 hosts, including 141 cattle breeds, 110 Asian water buffaloes, 43 sheep, and 56 goats, were observed during tick collection. The overall recorded tick occurrence was 64% (224/350). Based on the hosts, the highest occurrence of ticks was recorded in cattle breeds (104/141, 73.8%: Holstein-Friesian 54/57, 94.7%; Jersey 35/39, 89.7%; Sahiwal 12/17, 70.6%; and Achai 3/28, 10.7%) followed by Asian water buffaloes (68/110, 61.8%), goats (34/56, 60.7%), and sheep (18/43, 41.9%). The highest tick occurrence was recorded in the Peshawar district (239, 35.3%), followed by the Mardan district (183, 27.1%), Charsadda district (110, 16.3%), Swat district (52, 7.7%), Shangla district (48, 7.1%), and Chitral district (44, 6.5%) (Table 1). Different tick species, including *Rhipicephalus microplus*, *Rhipicephalus turanicus*, *Rhipicephalus haemaphysaloides*, *Hyalomma anatolicum*, *Hyalomma dromedarii*, *Hyalomma scupense*, *Haemaphysalis bispinosa*, *Haemaphysalis montgomeryi*, and *Haemaphysalis kashmirensis* were morphologically identified. Out of the total percentage composition for each tick species in all districts, the highest occurrence was observed for *R. microplus* (254, 37.6%), followed by *Hy. anatolicum* (136, 20.1%), *R. haemaphysaloides* (119, 17.6%), *R. turanicus* (116, 17.1%), *Ha. montgomeryi* (14, 2.1%), *Hy. dromedarii* (11, 1.6%), *Ha. bispinosa* (10, 1.5%), *Hy. scupense* (8, 1.2%), and *Ha. kashmirensis* (8, 1.2%). The most prevalent life stage of ticks was adult females (260, 38.5%), followed by nymphs (246, 36.4%) and adult males (170, 25.1%) (Table 1).

### 3.2. Variables Associated with a Tick Infestation

A total of 350 hosts of different ages, genders, and host types were observed for the presence of ticks in different seasons. Based on the hosts’ gender, the highest tick occurrence was noted in female hosts compared to male hosts. Based on age, hosts were stratified into three age groups; the highest tick occurrence was recorded in the <3 years age group, followed by the 1–3 years age group and >1-year age group. Among the observed animals, the highest occurrence was recorded in Holstein-Friesian, followed by Jersey, Sahiwal, Asian water buffaloes, goats, sheep, and Achai. The occurrence of ticks was highest in summer, followed by spring, fall, and winter seasons. District-wise, the occurrence of ticks was highest in the Peshawar district and lowest in Chitral. All variables, including genders, ages, hosts, seasons, and areas associated with tick occurrence, were highly significant (Table 2).

### 3.3. Detection of Anaplasma *spp.* in Ticks

The genomic DNA of 268 ticks (142N, 126F) was subjected to PCR to amplify the *16S rRNA* gene of *Anaplasma* spp. Out of 268 ticks, 22 (8.2%) (9/97 (9.2%) Peshawar, 6/74 (8.1%) Mardan, 4/46 (8.7%) Charsadda, 1/19 (5.2%) Swat, 1/15 (6.7%) Shangla, and 1/17 (5.9%) Chitral) were found positive for *Anaplasma* spp. Among various tick species, *R. microplus*, *R. turanicus*, and *R. haemaphysaloides* were found positive for *Anaplasma* spp. *Rhipicephalus microplus* ticks showed the highest occurrence for *Anaplasma* spp. (14, 63.6%), followed by *R. turanicus* (6, 27.3%), and *R. haemaphysaloides* (2, 9.1%). The district-wise occurrence of *Anaplasma* spp. was highest in Peshawar (9/97, 9.2%), followed by Charsadda (4/46, 8.7%), Mardan (6/74, 8.1%), Shangla (1/15. 6.7%), Chitral (1/17, 5.9%), and lowest in Swat (1/19, 5.2%). Positive *R. microplus*, *R. turanicus*, and *R. haemaphysaloides* infesting Holstein-Friesian and Jersey were reported from the Peshawar district, whereas positive *R. microplus*, *R. turanicus*, and *R. haemaphysaloides* infesting Holstein-Friesian, Jersey, and Asian water buffaloes were reported from the Mardan district. Furthermore, positive *R. microplus* infesting cattle breeds (Holstein-Friesian and Jersey) and Asian water buffaloes were reported from the Charsadda district. Positive *R. microplus* infesting Holstein-Friesian were reported from the Swat, Shangla, and Chitral districts (Table 1).

### 3.4. Phylogenetic Analysis of Anaplasma marginale

**Figure 2:**
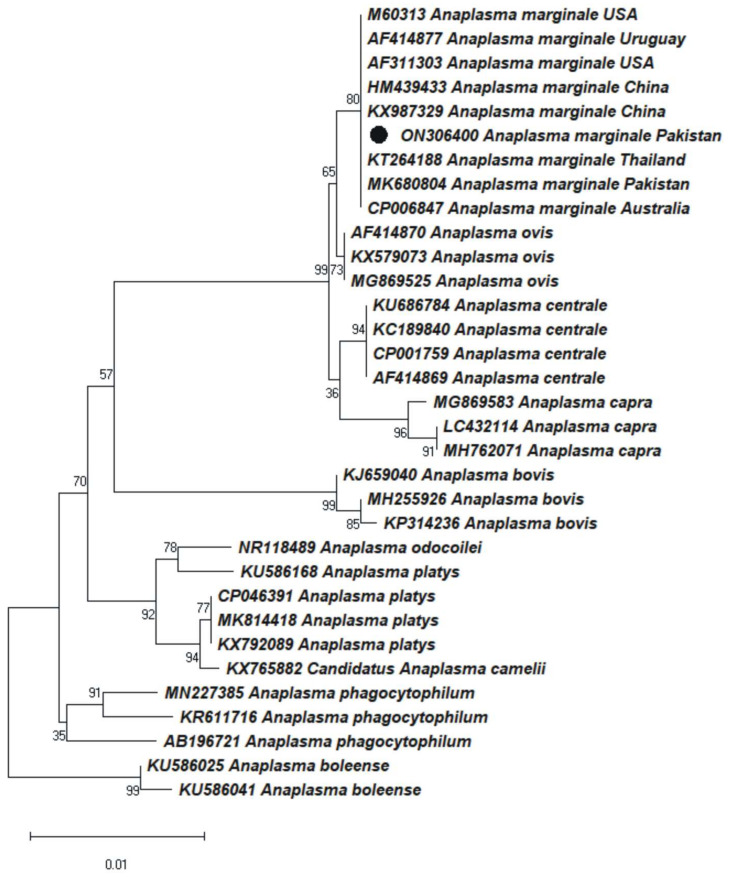
The maximum likelihood phylogenetic tree of *Anaplasma marginale* was constructed based on a partial *16S rDNA* sequence. *Anaplasma boleense 16S rDNA* sequences were used as an outgroup. The obtained sequence is represented with a black dot (ON306400).

The multiple obtained identical sequences were considered as a consensus sequence. The BLAST results of the obtained *16S rDNA* (1109 bp) sequences showed 98–100% identity with the *A. marginale* reported from Australia (CP006847 and AF414874), China (KX987330, DQ341369, FJ389579, and HM439433), Japan (FJ226454), Pakistan (MK680804, MK680806, and MK680807), Thailand (KT264188), Uganda (KU686785, KU686774, and KU686775), and the USA (AF311303, CP000030, and AF309866). Thirty-two sequences were downloaded for *16S rDNA* of *A. marginale* from NCBI. The phylogenetic tree obtained a *16S rDNA* sequence of *A. marginale* clustered with the identical species sequences reported from Australia, China, Thailand, Pakistan, Uruguay, and the USA (Figure 2). The *16S rDNA* sequence of *A. marginale* was uploaded to NCBI under the accession number ON306400.

## 4. Discussion

Pakistan’s hot and humid environment is ideal for the growth of ticks and their associated pathogens [5,21]. Previous studies have reported numerous tick species infesting diverse hosts in different areas of the country [5,15,18,20,35]. More than twenty different hard tick species are biological vectors of *A. marginale*, causing bovine anaplasmosis [36]. In Pakistan, there is a scarcity of molecular approaches to detecting *A. marginale* in ticks, primarily identified through microscopy of blood smear analysis [37,38]. During this study, *A. marginale* was genetically characterized in collected ticks from diverse livestock hosts in Pakistan. The current study identified hard ticks comprised of nine medically essential tick species infesting cattle breeds (Holstein-Friesian, Jersey, Sahiwal, and Achai), Asian water buffaloes, sheep, and goats. Among these nine tick species, three species in the genus *Rhipicephalus* were found positive for *A. marginale*, with *A. marginale* being dominant in *R. microplus*. This tick has been identified as a significant vector of *A. marginale* in several tropical and subtropical countries [39].

A low tick occurrence was recorded in local cattle breeds (Achai), which may be due to the natural resistance of Achai towards the tick infestation. The female hosts were found to have a significantly higher occurrence of ticks than male hosts, which is consistent with the previous findings [20]. Female hosts may have a high occurrence due to hormonal changes, because the high level of prolactin and progesterone hormone in female hosts make them more vulnerable to tick infestation [40]. A high tick occurrence has been recorded in adult hosts compared to the younger ones, which is in accordance with the findings of previous reports [17,35]. Free grazing practices and large surfaces of the adult hosts make them more susceptible to tick attachment, in contrast to the younger ones that get less of a tick burden due to less grazing practices, the low surface area of their bodies, and their strong immune system [41]. Annual patterns of tick activity are influenced by seasonal temperature variations, affecting the dynamics of ticks and TBDs. Fluctuation in different seasons may result in varying tick occurrence in the same region [42]. The summer season provides the best conditions for developing and expanding ticks. The winter season hampers the infestation of ticks because all stages of ticks hibernate in cold climatic conditions, and these findings support the previous reports in the region [15,35]. The highest tick occurrence was recorded in the Peshawar district, followed by Mardan, and the least was recorded from the Chitral district. These results may correspond to favorable environmental conditions associated with ticks rearing [18,35,43].

For identifying *Anaplasma* spp., molecular techniques such as PCR have significant advantages over traditional serological and blood smear testing because PCR is the most sensitive and reliable diagnostic method [44]. The molecular phylogeny of *A. marginale* from the study area was developed by amplifying *16S rDNA* sequences, as this marker is of prime importance in characterizing *Anaplasma* spp. [40]. The sequence obtained in this study shared a 98–100% identity with available sequences in GenBank. Phylogenetic analysis revealed that *A. marginale* from the northern regions of Pakistan clustered with related isolates reported from Australia, China, Thailand, Pakistan, Uruguay, and the USA (Figure 2). Previous research on the molecular phylogeny of *A. marginale* based on *16S rDNA* sequences from Pakistan validates our findings [20,45].

Ticks infected with *A. marginale* may be of significant concern to both animals and humans due to the increased risk of infection, complicating clinical care. These findings highlight the need for a larger-scale tick surveillance program in understanding various TBDs, and their zoonotic and pathogenic potential.

## 5. Conclusions

The present study provides information regarding the occurrence of hard ticks as carriers for *A. marginale* in Pakistan. *Anaplasma marginale* was detected in three tick species: *R. microplus*, *R. turanicus*, and *R. haemaphysaloides*. These findings will inform the veterinary and livestock community regarding the diversity of tick species and associated *A. marginale*. Further research is needed to explore the variety of ticks and tick-associated pathogens in Pakistan.

## Figures and Tables

**Table 1:** Occurrence of ticks in various hosts and molecular detection of *Anaplasma marginale* in different districts of Khyber Pakhtunkhwa, Pakistan.

District	Host	Observed Hosts	Infested Hosts (%)	Ticks	Tick Life Stages	Molecularly Screened Ticks	*Anaplasma* Positive Ticks
**Peshawar**	Holstein-Friesian	19	18 (94.7)	*R. microplus*	43 (19N, 16M, 8F)	15N, 8F	5N
				*R. turanicus*	21 (7N, 9M, 5F)	5N, 5F	2N
				*R. haemaphysaloides*	16 (8N, 3M, 5F)	3N, 5F	1N
				*Hy. scupense*	8 (6N, 2F)	1N, 1F	-
	Jersey	12	11 (91.6)	*R. microplus*	32 (15N, 10M, 7F)	9N, 7F	1F
				*Hy. anatolicum*	28 (4N, 4M, 20F)	1F	-
	Sahiwal	3	3 (100)	*R. microplus*	4 (2N, 1M, 1F)	1N, 1F	-
				*Hy. anatolicum*	21 (1N, 20F)	1F	-
	Achai	2	-	-	-	-	-
	Asian water buffaloes	21	16 (76)	*R. microplus*	12 (4N, 5M, 3F)	4N, 3F	-
				*R. turanicus*	7 (3N, 2M, 2F)	2N, 2F	-
				*R. haemaphysaloides*	33 (14N, 13M, 6F)	9N, 6F	-
	Sheep	13	7 (58.3)	*R. turanicus*	3 (1N, 1M, 1F)	1N, 1F	-
				*R. haemaphysaloides*	3 (1N, 1M, 1F)	1N, 1F	-
	Goats	11	7 (63.6)	*R. turanicus*	8 (3N, 4M, 1F)	3N, 1F	-
**Mardan**	Holstein-Friesian	16	15 (93.7)	*R. microplus*	8 (4N, 2M, 2F)	2N, 2F	2N
				*R. haemaphysaloides*	23 (11N, 7M, 5F)	7N, 5F	1N
	Jersey	10	9 (90)	*R. microplus*	8 (1N, 2M, 5F)	1N, 1F	-
				*R. turanicus*	21 (8N, 7M, 6F)	7N, 6F	1N
				*R. haemaphysaloides*	9 (4N, 3M, 2F)	2N, 2F	-
	Sahiwal	4	3 (75)	*R. microplus*	2 (1N, 1F)	1N, 1F	-
				*Hy. anatolicum*	16 (1M, I5F)	1F	-
	Achai	2	-	-	-	-	-
	Asian water buffaloes	21	14 (66.7)	*R. microplus*	31 (14N, 10M, 7F)	6N, 7F	-
				*R. turanicus*	21 (9N, 8M, 4F)	8N, 4F	2F
				*R. haemaphysaloides*	4 (2N, 1M, 1F)	1N, 1F	-
				*Hy. anatolicum*	26 (1N, 1M, 24F)	1F	-
	Sheep	10	5 (50)	*R. turanicus*	4 (2N, 1M, 1F)	1N, 1F	-
				*R. haemaphysaloides*	2 (1N, 1F)	1F	-
	Goats	10	7 (70)	*R. turanicus*	2 (1N, 1F)	1F	-
				*R. haemaphysaloides*	6 (2N, 2M, 2F)	2N, 2F	-
**Charsadda**	Holstein-Friesian	15	14 (93.3)	*R. microplus*	22 (10N, 7M, 5F)	8N, 5F	2N
				*Hy. dromedarii*	11 (6N, 4M, 1F)	1F	-
				*R. haemaphysaloides*	14 (6N, 3M, 5F)	5N, 3F	-
	Jersey	10	9 (90)	*R. microplus*	11 (5N, 3M, 3F)	5N, 1F	-
				*R. turanicus*	11 (4N, 3M, 4F)	3N, 1F	1F
				*Hy. anatolicum*	6 (2N, 2M, 2F)	1F	
	Sahiwal	4	2 (50)	*R. microplus*	2 (1N, 1F)	1F	-
				*Hy. anatolicum*	11F	1F	-
	Achai	2	-	-	-	-	-
	Asian water buffaloes	22	13 (59)	*R. microplus*	11 (5N, 3M, 3F)	4N, 2F	1F
	Sheep	8	2 (25)	*R. microplus*	2 (1N, 1F)	1F	-
	Goats	9	5 (55.5)	*R. microplus*	2 (1N, 1F)	1F	-
				*R. turanicus*	7 (3N, 2M, 2F)	2N, 1F	-
**Swat**	Holstein-Friesian	2	2 (100)	*R. microplus*	9 (4N, 3M, 2F)	2N, 1F	1N
				*R. haemaphysaloides*	2 (1N, 1F)	1F	-
	Jersey	3	2 (66.7)	*R. microplus*	4 (2N, 1M, 1F)	1N, 1F	-
				*R. haemaphysaloides*	2 (1N, 1F)	1F	-
	Sahiwal	1	1 (100)	*R. microplus*	3 (2N, 1F)	1F	-
	Achai	8	1 (12.5)	*R. microplus*	2(1N, 1F)	1F	-
	Asian water buffaloes	17	10 (58.8)	*R. microplus*	5 (2N, 1M, 2F)	2N, 1F	-
				*R. turanicus*	2 (1N, 1F)	1N, 1F	-
				*Hy. anatolicum*	9 (1N, 1M, 7F)	1F	-
	Sheep	4	1 (25)	*Ha. montgomeryi*	1F	1F	-
				*Ha. kashmirensis*	1F	1F	-
	Goats	9	6 (66.7)	*Ha. montgomeryi*	7 (3N, 3M, 1F)	1F	-
				*Ha. bispinosa*	5 (2N, 2M, 1F)	1F	-
**Shangla**	Holstein-Friesian	3	3 (100)	*R. microplus*	4 (2N, 1M, 1F)	1N, 1F	1N
				*Hy. anatolicum*	7F	-	-
				*R. turanicus*	7 (4N, 2M, 1F)	1N, 1F	-
	Jersey	2	2 (100)	*R. microplus*	3 (1N, 1M, 1F)	1F	-
				*R. turanicus*	2 (1M, 1F)	1F	-
	Sahiwal	3	2 (66.6)	*R. microplus*	2 (1N, 1F)	1F	-
	Achai	7	1 (14.3)	*R. microplus*	2 (1N, 1F)	1F	-
	Asian water buffaloes	16	10 (62.5)	*R. microplus*	14 (8N, 4M, 2F)	2N, 1F	-
				*R. haemaphysaloides*	2 (1N, 1F)	1F	-
	Sheep	3	1 (33.3)	*Ha. kashmirensis*	1F	1F	-
	Goats	9	4 (44.4)	*Ha. montgomeryi*	2 (1N, 1F)	1N	-
				*Ha. kashmirensis*	2 (1N, 1F)	1N	-
**Chitral**	Holstein-Friesian	2	2 (100)	*R. microplus*	4 (1N, 2M, 1F)	1N, 1F	1N
	Jersey	2	2 (100)	*R. microplus*	2 (1M, 1F)	1F	-
				*R. haemaphysaloides*	3 (1N, 2F)	1N	-
	Sahiwal	2	1 (50)	*R. microplus*	2 (1M, 1F)	1F	-
	Achai	7	1 (14.3)	*R. microplus*	2 (1N, 1F)	1N	-
	Asian water buffaloes	13	5 (38.46)	*Hy. anatolicum*	12 (1N, 11F)	1N	-
				*R. microplus*	6 (3N, 2M, 1F)	2N, 1F	-
	Sheep	5	2 (50)	*Ha. montgomeryi*	2 (1N, 1F)	1N	-
				*Ha. bispinosa*	5 (2N, 2M, 1F)	1N, 1F	-
	Goats	8	5 (62.5)	*Ha. montgomeryi*	2 (1M, 1F)	1F	-
				*Ha. kashmirensis*	4 (3N, 1F)	3N	-
**Overall total**		350	224 (64)		676 (246N, 170M, 260F)	268 (142N, 126F)	22 (8.2%)

**Table 2:** Tick infestation on the hosts’ gender, ages, hosts, year-round, and collection sites.

Variables	Levels		Total	Positive	X²	*p*-Value
Genders	Female		250	190	3.00	0.0125
	Male		100	34		
Ages	≤1 years		59	20	8.22	0.0164
	1–3 years		118	75		
	>3 years		173	129		
Hosts	Cattle Breeds	Holstein-Friesian	57	54	3.382	0.0024
		Jersey	39	35		
		Sahiwal	17	12		
		Achai	28	3		
		Total	141	104		
	Asian water buffaloes		110	68		
	Sheep		43	18		
	Goats		56	34		
Seasons	Spring		105	71	3.227	0.0009
	Summer		140	101		
	Fall		55	31		
	Winter		50	21		
Areas	Peshawar		81	62	0.8165	0.0001
	Mardan		73	53		
	Charsadda		70	45		
	Swat		44	23		
	Shangla		43	23		
	Chitral		39	18		

## Data Availability

Details regarding data supporting reported results can be found https://www.ncbi.nlm.nih.gov/nuccore/?term= (accessed on 18 March 2022).
